# Systematic review of prediction models in relapsing remitting multiple sclerosis

**DOI:** 10.1371/journal.pone.0233575

**Published:** 2020-05-26

**Authors:** Fraser S. Brown, Stella A. Glasmacher, Patrick K. A. Kearns, Niall MacDougall, David Hunt, Peter Connick, Siddharthan Chandran

**Affiliations:** 1 Anne Rowling Regenerative Neurology Clinic, University of Edinburgh, Edinburgh, United Kingdom; 2 Institute of Neurological Sciences, Glasgow, United Kingdom; 3 MRC Institute of Genetics and Molecular Medicine, University of Edinburgh, Edinburgh, United Kingdom; 4 Centre for Clinical Brain Sciences, University of Edinburgh, Edinburgh, United Kingdom; 5 UK Dementia Research Institute, University of Edinburgh, Edinburgh, United Kingdom; University of Oxford, UNITED KINGDOM

## Abstract

The natural history of relapsing remitting multiple sclerosis (RRMS) is variable and prediction of individual prognosis challenging. The inability to reliably predict prognosis at diagnosis has important implications for informed decision making especially in relation to disease modifying therapies. We conducted a systematic review in order to collate, describe and assess the methodological quality of published prediction models in RRMS. We searched Medline, Embase and Web of Science. Two reviewers independently screened abstracts and full text for eligibility and assessed risk of bias. Studies reporting development or validation of prediction models for RRMS in adults were included. Data collection was guided by the checklist for critical appraisal and data extraction for systematic reviews (CHARMS) and applicability and methodological quality assessment by the prediction model risk of bias assessment tool (PROBAST). 30 studies were included in the review. Applicability was assessed as high risk of concern in 27 studies. Risk of bias was assessed as high for all studies. The single most frequently included predictor was baseline EDSS (n = 11). T2 Lesion volume or number and brain atrophy were each retained in seven studies. Five studies included external validation and none included impact analysis. Although a number of prediction models for RRMS have been reported, most are at high risk of bias and lack external validation and impact analysis, restricting their application to routine clinical practice.

## Introduction

The natural history of relapsing remitting multiple sclerosis (RRMS) is variable and prediction of individual prognosis is challenging [1]. The inability to reliably prognosticate at diagnosis has important implications for informed decision making especially in relation to disease modifying therapy (DMT). Risk stratification at diagnosis into disease severity categories (mild, moderate or severe) could better allow treating physicians and people with RRMS to make treatment decisions, but this is difficult early in the disease process.

As a consequence, broadly speaking, there are two treatment strategies in early RRMS: induction and escalation [1]. An induction strategy involves initiation of potent DMTs early in disease course[1]. An escalation strategy, on the other hand, involves initiating therapy with less potent agents with lower risk of serious adverse reactions, and then subsequently offering escalation to more potent DMTs if necessary. The induction strategy offers early control of disease but may cause harm from overtreatment. The escalation strategy risks harm from undertreatment and preventable neuroinflammation. As RRMS disproportionately affects individuals of working age, including females of childbearing potential, often pragmatic decisions need to be made that fall between these two strategies. For many reasons, therefore, there is a need for predictive tools that can be used by individual patients to inform treatment and life choices [2,3].

Multiple individual clinical and paraclinical factors have been studied for their ability to discriminate between patients with differing short and long-term prognoses. Poor prognosis has been associated with male sex and older age at disease onset [1,2]. However, a systematic review identified that evidence supporting the former is poor while predictive effect of older age is dependent on its definition [2]. Early clinical features such as sphincter involvement, higher baseline disability [2,4–7] and certain magnetic resonance imaging (MRI) measures- brain atrophy rate and T2-weighted lesion number and volume[8–12]- appear to be the most robust predictors of poor prognosis but these rely on established damage and so are not ideal prognostic measures. In contrast, biomarkers such as vitamin D level may confer prognostic effect at an earlier time point: an inverse relationship between serum vitamin D levels and hazard of relapse at six months has been reported [13]. The presence of cerebrospinal fluid (CSF) immunoglobulin M oligoclonal bands (IgMOB) is a putative biomarker for future relapse and conversion to secondary progression in RRMS, but requires further validation [14,15]. Lifestyle factors have attracted attention as they are potentially modifiable: Smoking has been shown to shorten time to onset of secondary progression [16,17]. However, whilst obesity appears to increase the chances of developing multiple sclerosis, its role in determining prognosis remains to be determined [18].

In a previous systematic review Langer-Gould et al focused on individual clinical and demographic factors in RRMS rather than composite models and did not include imaging variables [2]. Havas et al reviewed prediction models in RRMS focusing on predicting treatment response [19]. Predictive modelling, using patient-specific data points to predict outcome, is an unmet need in RRMS. Using published guidance for reporting and risk of bias assessment, our systematic review aims to add to this literature by describing and evaluating the methodological quality of studies that develop and validate predictive models in RRMS.

## Methods

Review aim, scope, target population, outcomes and intended moment of model use were defined as guided by CHARMS [20 and S1 File]. Study details and pre-specified search strategy were registered through PROSPERO, reference CRD42019149140 (https://www.crd.york.ac.uk/prospero/).

This review reports on studies that identify predictors of target outcomes, assign weights (eg. using regression coefficients) to each predictor using multivariable analysis, and develop a prediction model for adult patients with RRMS. Herein, a prediction model is taken to mean a model which uses multiple predictors in combination to determine probability of an outcome [23]. Intended moment of model use is at diagnosis. External validation studies were also included. We excluded studies predominantly selecting children (<18 years old), predicting response to disease modifying therapy, predicting conversion of clinically isolated syndrome (CIS) to MS, studies exclusively including patients with CIS, primary progressive multiple sclerosis (PPMS), secondary progressive multiple sclerosis (SPMS) or studies investigating a single predictor, test or marker as they do not meet the definition of prediction model as above. Outcomes of interest included inflammatory disease activity (clinical relapse rate, T2 lesion load change), rate of neurodegeneration (brain atrophy, clinical progression of fixed disability), progression to SPMS and degree of disability.

A search of OVID MEDLINE, Embase and ISI Web of Science was conducted using a pre-specified search strategy [S2 File]. Records not meeting inclusion criteria or clearly not prediction modelling studies were excluded by one reviewer. The remaining records were screened by two medically qualified reviewers [FSB and SAG] independently and full articles were reviewed if eligible. Disagreements were resolved by consensus. There were no limitations with regard to study language or publication date.

Data extraction (following CHARMS [20]) was performed by one reviewer and quality assessment by two reviewers. The categories for data extraction are detailed in full in the PROSPERO record but include source of data, participants, outcome candidate predictors, model development and model evaluation. Quality assessment of studies was carried out following Prediction model Risk Of Bias ASsessment Tool [PROBAST]) which rates study methodology and applicability to review question as at “high”, “low” or “unclear” risk of bias based on a predetermined set of questions and scoring guide [21]. Inter-rater agreement in these domains was measured by Cohen’s kappa statistic.

## Results

**Fig 1:**
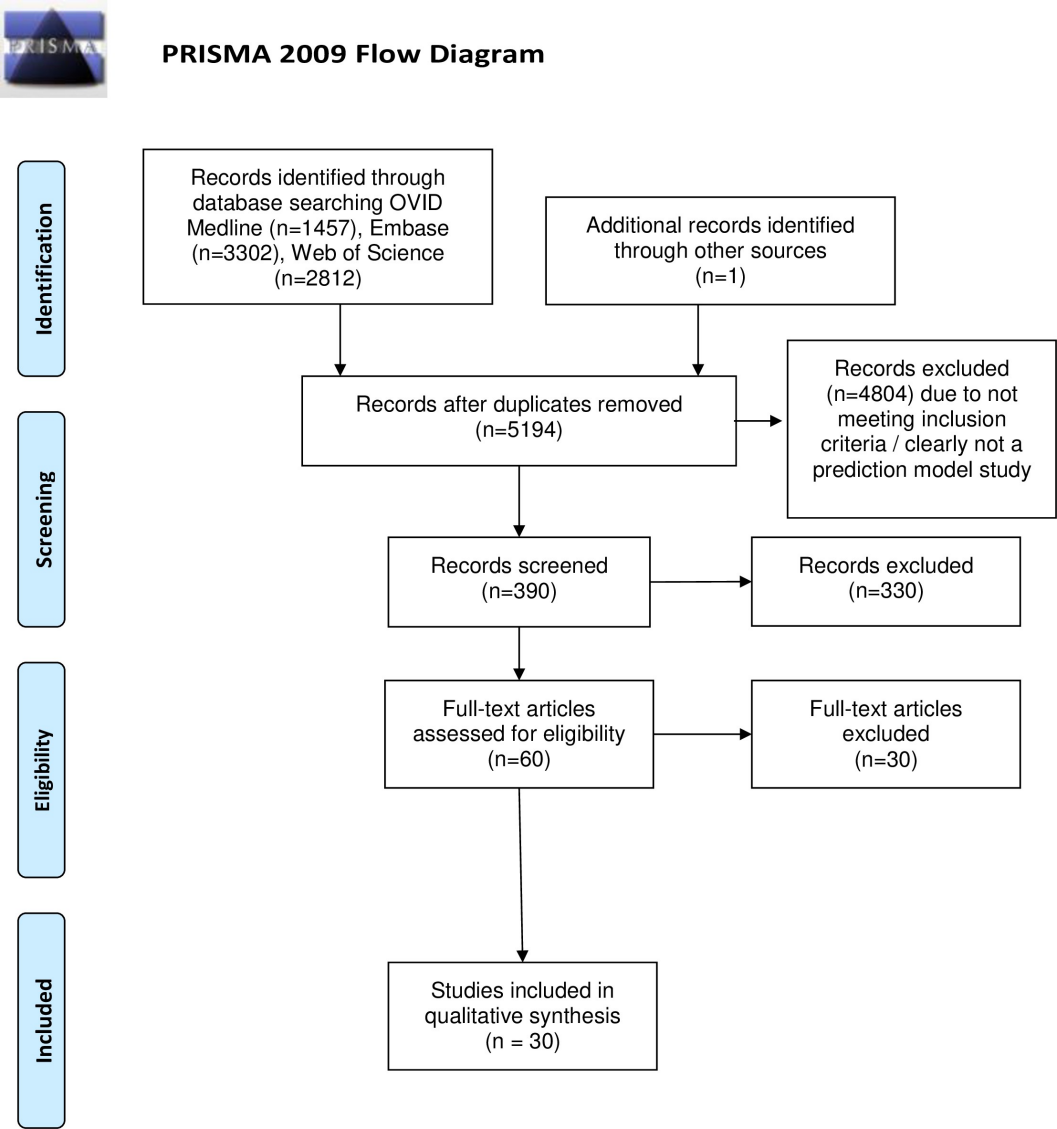
PRISMA flow diagram.

Database searches from inception to August week three 2019 identified 5193 studies of which 30 studies met the pre-defined inclusion criteria (Fig 1) [22–51]. 23 studies were model development only [22–24,26,29–34,37,39–46,48–51], five were model development and external validation in the same study [25,35,36,38,47] and two were external validation studies of the same model [27,28]. Studies used Poser (n = 16), McDonald 2001 (n = 4), McDonald 2005 (n = 6) and McDonald 2010 (n = 2) diagnostic criteria (S3 File). Diagnostic criteria were not specified in five studies [23,32,35,36,51]. Four studies used more than one set of diagnostic criteria [22,31,37,38]. Two studies used data from multiple clinical trials likely with heterogeneous diagnostic criteria [24,33]. A summary of study attributes is included in S4 File and risk of bias assessment in Table 1. Agreement in PROBAST assessment between reviewers was 96.5% and 79.3% for overall concern for risk of bias and applicability to our research question, respectively.

**Table 1:** PROBAST: Assessment of risk of bias and applicability of a) development and b) external validation papers.

**a**							
Study	ROB				Applicability		
	Participants	Predictors	Outcome	Analysis	Participants	Predictors	Outcome
Agosta 2006	✖	✔	✔	✖	✖	✖	✔
Bakshi 2008	✖	✔	✔	✖	✖	✖	✔
Barkhof 2005	✖	✖	✖	✖	✖	✔	✔
Bejarno 2011	✖	✔	✔	✖	✖	✖	✔
Bergamaschi 2001	✖	✔	✖	✖	✖	✔	✔
De Groot 2009	✖	✖	✖	✖	✖	✔	✔
Dekker 2019	✖	✔	✖	✖	✖	✖	✔
Filippi 2012	✖	✔	✖	✖	✖	✖	✔
Gauthier 2007	✔	✔	✖	✖	✖	✖	✔
Held 2005	✖	✖	✖	✖	✖	✖	✔
Liguori 2011	✖	✖	✖	✖	✖	✖	✔
Mandrioli 2008	✖	✖	✖	✖	✖	✔	✔
Manouchehrinia 2019	✖	✔	✖	✖	✔	✔	✔
Margaritella 2012 (A)	✖	✖	✖	✖	✖	✖	✔
Margaritella 2012 (B)	✖	✔	✖	✖	✖	✖	✔
Mesaros 2008	✖	**?**	✖	✖	✔	✖	✔
Minneboo 2008	✖	✔	✖	✖	✖	✔	✔
Popescu 2013	✖	✔	✖	✖	✖	✖	✔
Ramsaransing 2007	✖	✖	✖	✖	✖	✖	✔
Runmarker 1994	✖	✖	✖	✖	✔	✔	✔
Schlaeger 2012	✖	✔	✔	✖	✖	✖	✔
Schlaeger 2014	✖	✔	✔	✖	✖	✖	✔
Skoog 2014	✖	✖	✖	✖	✔	✖	✔
Sormani 2007	✖	✔	✖	✖	✖	✖	✔
Uher 2017	✖	✔	✖	✖	✖	✖	✔
Von Gumberz 2016	✖	✔	✖	✖	✔	✖	✔
Weideman 2017	✖	✔	✖	✖	**?**	✖	✖
Weinshenker 1991	✖	✖	✖	✖	✖	✔	✔
**b**							
Study	ROB				Applicability		
	Participants	Predictors	Outcome	Analysis	Participants	Predictors	Outcome
Bergamaschi 2007	✖	✖	✖	✖	✖	✔	✔
Bergamaschi 2015	✖	✖	✖	✖	✔	✔	✔

PROBAST assessment performed by two independent reviewers with Kappa value 0.458. Final agreed assessment presented. Where more than one model was developed in a study, PROBAST scoring is reported only once. ✔ = low risk of bias, ✖ = high risk of bias,**? =** unclear risk of bias.

### Source of data and participants

27 studies [22,23,25–32,34–46,48–51] used cohort design, which is recognised as an optimal strategy for prediction model development. Three studies used data from clinical trials [24,33,47]. 21 studies were single centre and nine multicentre. All 30 studies reported inclusion and exclusion criteria. 11 studies featured populations consisting only of patients with RRMS [27,28,35,36,38,39,43,46–49]. Percentage of patients treated with DMTs in studied cohorts varied from 0–100% (S5 File). Only four studies were judged not to be at high risk of selection bias [28,39,42,47].

### Candidate predictors

Demographic, clinical, MRI, CSF and electrophysiology variables were retained as predictors in final models (Table 2). The single most common clinical predictor was baseline EDSS (n = 11). Age (n = 6), age at onset (n = 6) and gender (n = 5) were also commonly retained. T2 lesion volume or number and brain atrophy were each retained in seven studies. In ten studies, predictor measurement timing matched our review question’s target timing (that is, the authors studied variables present at time of diagnosis with RRMS) [24,26–29,35,36,40,50,51]. In nine studies, subjective predictor definitions or variable determination methods were used [24,29,33–35,37,43,46,51]. In nine studies, continuous predictors were categorised, another potential source of bias [26–28,32–34,36,44,48]

**Table 2:** Frequency of variables included in prediction models by development study.

Study	Variable								
	Age	Onset age	Gender	Clinical	MRI	EP	CSF	FMHx	DMT
Agosta 2006					✔				
Bakshi 2008				✔	✔				
Barkhof 2005		✔		✔	✔				
Bejarno 2011				✔		✔			
Bergamaschi 2001		✔	✔	✔					
De Groot 2009				✔	✔				
Dekker 2019				✔	✔				
Filippi 2012					✔				
Gauthier 2007	✔			✔	✔				
Held 2005				✔					
Liguori 2011					✔				
Mandrioli 2008				✔			✔		
Manouchehrinia 2019	✔	✔	✔	✔					
Margaritella 2012 (A)		✔	✔	✔		✔			
Margaritella 2012 (B)				✔		✔			
Mesaros 2008				✔					
Minneboo 2008	✔			✔	✔				
Popescu 2013					✔				
Ramsaransing 2007				✔					
Runmarker 1994		✔	✔	✔					
Schlaeger 2012					✔				
Schlaeger 2014						✔			✔
Skoog 2014	✔			✔					
Sormani 2007				✔	✔				
Uher 2017				✔	✔				
Von Gumberz 2016				✔	✔				✔
Weideman 2017	✔			✔				✔	✔
Weinshenker 1991	✔	✔	✔						

MRI: magnetic resonance imaging. EP: electrophysiology. CSF: cerebrospinal fluid. FMHx: Family history. DMT: disease modifying therapy.

### Model outcomes

Three studies had outcomes which were not objectively defined [34,43,46]. Within studies, the same outcome assessment method was generally applied to all patients. In four studies using EDSS as an outcome measure different assessment methods (telephone EDSS as opposed to full examinations) were used in some patients [31,41,44,45]. External validation in one study used a different definition of secondary progression to the development cohort [36]. No studies reported blinding of outcome assessors to all predictor information.

### Model development and evaluation

Regression analysis was the most common modelling technique (n = 24). Neural networks, gradient boosting machine, Bayesian and Markov modelling techniques were each used in one model development study. Ten studies used univariate or bivariate analyses to filter potential predictors. Outcome events per predictor ratio (EPV) of less than 10 is a widely recognised criterion for identifying models at risk of overfitting [21]. This calculation is not applicable to models with continuous outcomes. There was insufficient information to calculate EPV in four studies [32,37,43,47]. 12 of the 16 studies in which EPV was applicable and could be calculated had scores of <10 (S6 File). 18 studies used complete case analysis. Four studies reported imputation: three used last observation carried forward and one used multiple imputation. In eight studies there was insufficient information to determine missing data handling.

Internal validation was present in nine studies: four used cross-validation, three used split-sample and two used bootstrap. Only one study reported applying shrinkage methods [29]. Discrimination and calibration are common prediction model performance measures [21]. Discrimination is commonly assessed by area under the receiver operator curve (AUC) while it is recommended calibration be presented as a plot of observed versus predicted outcomes [21]. AUC was reported in ten studies and ranged from 0.64 to 0.89. Calibration was reported graphically in four studies. Goodness of fit performance statistics R^2^ or nagelkerke R^2^ were reported in seven studies.

Five studies included external validation in their model development. In two instances, this was restricted to temporal external validation [35,38]. Only one study reported performance measures in the external validation cohort where AUC ranged between 0.77–0.87 [35]. The Bayesian Risk Estimate for MS score [26] was externally validated in two subsequent studies with the second removing predictors [29,30]. AUC was not reported in either of the BREMS validation cohorts [29,30]. All external validation was performed by the authors of the respective development models.

### Presentation of model and utility

Five studies presented the outcome of model development as a risk score [26–29, 48]. Two presented a web-based application [46,50]. One presented a nomogram [36]. For example, Skoog et al produced an online prediction score calculator on a freely available website which allows input of the current age of the patient, time since most recent attack, the main symptom type and whether there has been complete remission of most recent attack [46]. The output of this score is a percentage annual risk of conversion to secondary progression [46]. None of the studies carried out an impact assessment.

## Discussion

Here, we present a systematic review of studies investigating prognostic models for use in people with RRMS. In the models studied, the single most common clinical predictor was baseline EDSS (n = 11). Demographic variables, including age and sex, and MRI markers, including T2 lesion volume or number and brain atrophy, were also often retained in the models studied here. Only one study included a CSF marker- IgMOB- in its final model. Vitamin D levels and smoking status, which have some published support for their prognostic relevance, did not feature in any models.

Our results demonstrate that there is agreement between a limited number of studies showing the prognostic effects of demographic and radiological parameters [8–12, 19]. We identified no studies that incorporated demographic, radiological, and biomarker data. Other work, including reviews by Langer-Gould et al (focusing on individual predictors) and Havas et al (focusing on predicting treatment response) also identified early disease course as a predictor of outcome [2,4–6,19].

### Applicability

Most of the models studied were not developed and / or validated for use at time of diagnosis of RRMS. In addition, this cross section of newly diagnosed patients has changed over time with the evolution of diagnostic criteria [52]. None of the models were developed or validated in cohorts whose diagnosis was made using the 2017 McDonald criteria.

Many predictors required information unavailable at the time of diagnosis such as longitudinal disease course features. Optimally, a prediction tool would be applicable at time of diagnosis to facilitate initial treatment decisions and would incorporate predictively relevant information from all domains that are available at that point. That is, to tailor a treatment strategy for a patient that falls between the escalation and initiation strategies based on a best estimation of that individual’s risks. Furthermore, the majority of studies included patients taking DMTs. Variable DMT usage introduces heterogeneity between studies and between participants within studies, and therefore hampers interpretation and comparisons. An improved understanding of the impact of DMTs on long-term outcomes will be needed in order to fully inform model-guided treatment strategies. As such, there is still a major unmet need with regard to developing prediction models applicable to patients with newly diagnosed RRMS.

### Risk of bias

All included studies were at an overall risk of bias. Selection bias was a concern in the majority of studies. Often this was due to exclusion or inappropriate imputation of participants with missing data. The majority of studies used complete case analysis which can introduce bias given potential non-random distribution of missing data [21]. In addition, the last visit carried forward approach will flatter participants who are lost to follow up. Further selection bias was judged likely due to inclusion being limited to non-representative subgroups of the RRMS population. Predictor determination and definitions were subject to variability in some studies meaning associations with outcome may not be generalizable. Blinding of outcome assessors to predictor information was poorly reported. Blinding outcome assessors to predictor information is especially for preventing bias when assessments are subjective and require interpretation, as is the case with many of the clinical outcomes employed here [21, 53].

Small sample sizes were common which limited the power of many models to examine multiple parameters or interactions between parameters [54]. Univariable analysis was often used to select predictors for model inclusion, which risks omitting predictors with important relationships with the outcome present only after adjustment for confounding covariates, and risks inclusion of covariates that hold no independent predictive power when other covariates are included [21, 55]. Model calibration was poorly reported. AUC- a measure of discrimination- was reported only in ten studies. Reported AUC values in ten model development studies ranged between 0.64–0.89 (0.7–0.8 is regarded as acceptable and 0.8–0.9 excellent [56]). Without reporting of calibration and discrimination it is challenging to quantify model accuracy [21].

The majority of studies did not perform internal validation. Models without internal validation may be at risk of misspecification (e.g. overfitting to development data sets) [57]. External validation was only reported in three studies [35,36,38] and performance statistics were only presented in one of these [36]. Lack of reporting of external validation and model performance therein undermines use of model in different patients [58]. No studies performed impact analysis, an essential step which quantifies changes in clinician behaviour, outcomes and cost-effectiveness of implementing models and provides an evidence base for clinical practice [59]. None of the studies incorporated clinical, radiological, demographic, lifestyle and biomarker predictors though independently, each of these has been demonstrated to show predictive power. As such, PROBAST assessment has identified areas for improvement in order to limit risk of bias in future studies.

In summary, issues of applicability and methodological quality limit the application of the studied models.

### Future perspectives

The present study does not investigate fatigue and cognitive impairment as outcomes. These symptoms are increasingly recognised as contributors to morbidity in MS [60]. Inclusion of these factors was beyond the scope of this review but they should be researched further and may be worthy of inclusion in future attempts to construct predictive models. An improved understanding of the underlying pathobiological and molecular mechanism(s) of MS is likely to lead to a range of biomarkers that may feature in future predictive models. Differential gene transcription levels have been shown to predict interferon beta responsiveness in RRMS [61]. RNA profiling can identify patients with different levels of disease activity [62]. Single Molecule Array (SIMOA) technology offers increasingly accurate quantification of biomarkers such as neurofilament light chain [63, 64]. Imaging measures also show promise. Atrophied T2 lesion volume, a result of both inflammatory and degenerative processes, has been identified as a predictor of future disease activity in RRMS [65]. Ultra-high field (7 tesla) MRI shows promise in longitudinal investigation of multiple sclerosis lesions [66].

For individuals with newly diagnosed RRMS, reliable prognostic models are urgently needed. However, with a growing number of promising biomarkers, improvements in capabilities in novel imaging techniques, and increased understanding of the demographic, clinical, and immunological basis of MS heterogeneity, large well-powered cohorts will be necessary in order to have sufficient power to combine these predictive modalities into clinically useful tools. Persons newly diagnosed with RRMS face uncertainty regarding future disease course and the effect of treatment. Methodologically sound models developed in appropriate patient populations are vital to improve prognostication and inform therapeutic decision-making.

## Supporting information

S1 Checklist (DOC)

S1 File CHARMS protocol. Design of the systematic review based on Critical Appraisal and Data Extraction for Systematic Reviews of Prediction Modelling Studies: The CHARMS Checklist.
(DOCX)

S2 File Search strategy. (DOCX)

S3 File Diagnostic criteria. (DOCX)

S4 File CHARMS data extraction. (DOCX)

S5 File Percentage of study participants on DMTs. (DOCX)

S6 File Events per variable per study and calculation method. (DOCX)
