# Supplementary material for: Systematic review of prediction models in relapsing remitting multiple sclerosis
Source: PLoS One. 2020 May 26;15(5):e0233575. doi: 10.1371/journal.pone.0233575 (PMC7250448; doi:10.1371/journal.pone.0233575)
Supplement: S1 File — Design of the systematic review based on Critical Appraisal and Data Extraction for Systematic Reviews of Prediction Modelling Studies: The CHARMS Checklist. (DOCX) [file pone.0233575.s002.docx]

**S1 File. CHARMS Protocol.** Design of the systematic review based on Critical Appraisal and Data Extraction for Systematic Reviews of Prediction Modelling Studies: The CHARMS Checklist.

| 1. Prognostic versus diagnostic prediction model | The aim is to review models to predict future events in relapsing remitting multiple sclerosis (RRMS). |
| --- | --- |
| 1. Intended scope of the review | Review of all published models for predicting prognosis in RRMS. Both model development and external validation studies will be included. The review will exclude models designed solely for use in clinically isolated syndrome or progressive multiple sclerosis. |
| 1. Type of prediction modelling studies | Prediction model development with or without external validation in independent data. |
| 1. Target population to whom the prediction model applies | Adult patients (>/= 18 years) diagnosed with RRMS. |
| 1. Outcome to be predicted | 1. Inflammatory disease activity:  (i) Clinical relapse rate  (ii) T2 lesion load change 2. Rate of neurodegeneration: 3. Brain atrophy 4. Clinical progression of fixed disability 5. Progression to secondary progressive multiple sclerosis. 6. Degree of disability. |
| 1. Time span of prediction | Indefinite |
| 1. Intended moment of using the model | Models to be used at the moment of diagnosis of RRMS and early disease to predict prognosis. |
