# Supplementary material for: Systematic review of prediction models in relapsing remitting multiple sclerosis
Source: PLoS One. 2020 May 26;15(5):e0233575. doi: 10.1371/journal.pone.0233575 (PMC7250448; doi:10.1371/journal.pone.0233575)
Supplement: S2 File — (DOCX) [file pone.0233575.s003.docx]

**S2 File. Search strategy**

***Database: Ovid MEDLINE(R)***

Limited to human studies.

1. exp multiple sclerosis/

2. (risk adj3 (scor*or assess* or apprais* or equation or calculate* or model$ or predict$

or tool$)).ti,ab.

3. model$.ti.

4. predict*.ti.

5. algorithm$.ti.

6. scoring scheme.ti.

7. or/2-6

8. 1 and 7

***Database: Ovid EMBASE(R)***

Limited to human studies.

1. exp multiple sclerosis/

2. (risk adj3 (scor*or assess* or apprais* or equation or calculate* or model$ or predict$

or tool$)).ti,ab.

3. model$.ti.

4. predict*.ti.

5. algorithm$.ti.

6. scoring scheme.ti.

7. or/2-6

8. 1 and 7

***Database: Web Of Science Core Collection***

No conference abstracts. By title.

(multiple sclerosis) and (risk or predict* or model)

Performed on 03/09/2019.
