# Supplementary material for: Systematic review of prediction models in relapsing remitting multiple sclerosis
Source: PLoS One. 2020 May 26;15(5):e0233575. doi: 10.1371/journal.pone.0233575 (PMC7250448; doi:10.1371/journal.pone.0233575)
Supplement: S3 File — (DOCX) [file pone.0233575.s004.docx]

**S3 File. Diagnostic criteria**

| Study | Criteria |
| --- | --- |
| Agosta 2006 | Poser and McDonald (2005) |
| Bakshi 2008 | Definitive diagnosis- CLIMB study |
| Barkhof 2005 | Data from 17 RCTs |
| Bejarno 2011 | McDonald (2005) |
| Bergamaschi 2001 | Poser |
| Bergamaschi 2007 | Poser |
| Bergamaschi 2015 | McDonald 2001 |
| De Groot 2009 | Poser |
| Dekker 2019 | McDonald 2005 |
| Filippi 2012 | Poser, McDonald 2001 |
| Gauthier 2007 | Definitive diagnosis- CLIMB study |
| Held 2005 | 22 RCTs |
| Liguori 2011 | McDonald 2005 |
| Mandrioli 2008 | Clinically definite MS |
| Manouchehrinia 2019 | Large longitudinal database, likely not uniform |
| Margaritella 2012 (A) | McDonald 2001, 2005 and Poser |
| Margaritella 2012 (B) | Poser, McDonald 2001 |
| Mesaros 2008 | Poser |
| Minneboo 2008 | Poser |
| Popescu 2013 | McDonald 2005 |
| Ramsaransing 2007 | Poser |
| Runmarker 1994 | Poser |
| Schlaeger 2012 | Poser |
| Schlaeger 2014 | Poser |
| Skoog 2014 | Poser |
| Sormani 2007 | Poser |
| Uher 2017 | Poser |
| Von Gumberz 2016 | McDonald 2010 |
| Weideman 2017 | McDonald 2010 |
| Weinshenker 1991 | Not specified |
