# Supplementary material for: Systematic review of prediction models in relapsing remitting multiple sclerosis
Source: PLoS One. 2020 May 26;15(5):e0233575. doi: 10.1371/journal.pone.0233575 (PMC7250448; doi:10.1371/journal.pone.0233575)
Supplement: S4 File — (DOCX) [file pone.0233575.s005.docx]

**S4 File. Study characteristics.**

| **Study** | **Design**  **and Study Population Size** | **Model Outcome** | **Candidate Variables** | **Method of Predictor Selection** | **Included Predictor Variables** | **Missing Data** | **Modelling Method** | **Discrimination (c-index or AUC)**  **Calibration Validation** | **Presentation** |
| --- | --- | --- | --- | --- | --- | --- | --- | --- | --- |
| Agosta *et al.* 2006 | Cohort (P)  Single centre (n=73) | Worsening disability | 25 | Pre-selection by univariate analysis then multivariate analysis. P-value<0.2 for univariate analysis | Baseline GM histogram peak height and average lesion MTR percentage change after twelve months. |  | Multivariate logistic regression | Discrimination: absent.  Calibration: absent  Internal validation: cross-validation  External validation: absent | None |
| Bakshi *et al.* 2008 | Cohort (P)  Single centre (n=103) | Worsening disability | 6 | Clinical reasoning used to select predictors | Composite score of: 1) T2 lesion volume 2) Brain parenchymal fraction 3) T1:T2 ratio and baseline EDSS | Complete case analysis | Multivariate logistic regression | Discrimination: AUC 0.68. Calibration: absent  Validation: absent | None |
| Barkhof *et al.* 2005 | Placebo arms of clinical trials  Multicentre  (n=1328) | Disease activity (gadolinium enhancing lesions) | 9 | Pre-selection by univariate analysis then multivariate analysis. P-value <0.05 for multivariate analysis. | Subset 1: disease course, age at onset and disease duration. Subset 2: T2 disease burden, disease course, disease duration and age at disease onset. | Complete case analysis | Multivariate logistic regression | Discrimination: AUC Subset 1: 0.64, Subset 2: 0.74.  Calibration: absent  Internal validation: split sample  External validation: absent | None. |
| Bejarno *et al.*2011 | Cohort (P)  Single centre  (n=51) | Degree of disability, worsening disability | 19 | Algorithmic ranking of predictive ability, highest ranked entered classifiers for neural network | EDSS at baseline, CMCT and MEP. |  | Neural network | Discrimination: AUC: 0.76. Calibration: absent.  Internal validation: cross-validation.  External validation: overall accuracy reported as 80% | None. |
| Bergamaschi *et al.* 2001 | Cohort (R)  Single centre  (n=186) | Onset of secondary progression | 17 | Monte Carlo particle filtering | Onset: sphincter, pure motor or motor/sensory sequelae of onset, number of functional systems involved, sphincter and motor relapses in year 1, EDSS>4 outside relapse year 1, gender, age at onset. |  | Bayesian Model | Discrimination: absent  Calibration: absent. No internal validation. | Risk score |
| Bergamaschi *et al.* 2007  (External validation) | Cohort (R)  Multicentre  (n=1245) | Onset of secondary progression | NA | NA | Age at onset, gender, sphincter onset, pure motor onset, motor-sensory onset, sequelae after onset, functional systems involved at onset, sphincter plus motor relapses, EDSS>4 outside relapse. |  | NA | Discrimination: absent  Calibration: absent  >95^th^ percentile: specificity 0.99, sensitivity 0.17, PPV 0.86 and NPV. <5th percentile remained progression free at 10 years: specificity 1.00, sensitivity 0.08, PPV 1.00, NPV 0.18. | Risk score |
| Bergamaschi *et al.* 2015  (Exernal validation) | Cohort (P) Multicentre  (n=14211) | Onset of secondary progression | NA | NA | Age at onset, gender, sphincter onset, pure motor onset, motor-sensory onset, number of neurological functional systems involved at onset, incomplete recovery after onset |  | NA | Statistically significant differences between survival curves in <1st quartile and >3rd quartile. | Risk score |
| De Groot *et al.* 2009 | Cohort (P)  Multicentre  (n=146) | Disability | 10 | Backwards stepwise selection multivariate logistic regression P-value<0.5. | Self-reported walking ability, cerebellar impairment, spinal cord lesion on MRI | Imputation | Multivariate logistic regression | Discrimination:  AUC: 0.89.  Calibration:  Calibration curves good fit. Internal validation: Bootstrap and shrinkage methods applied.  External validation: absent | Risk score |
| Dekker *et al.* 2019 | Cohort (P)  Single centre  (n=115) | Disability | 18 | Pre-selection by univariate analysis P-value <0.1 then backward selection multivariate linear regression P-value<0.1. | For year 6: baseline-year 2 EDSS change, PPMS, early brain atrophy. Year 12: baseline-year 2 EDSS, baseline T1LV. |  | Multivariate linear regression | Discrimination: C-statistic NA to continuous outcome  Calibration: absent  Validation: absent |  |
| Filippi *et al.* 2013 | Cohort (P)  Single centre  (n=73) | 1: benign MS  2: onset of secondary progression  3: Progression from CIS to RRMS, RRMS to SPMS or from SPMS to death | 29 | Random forest analysis ranked candidate predictors then stepwise selection multivariate logistic regression | 1: Baseline: disease duration, GMF. 12 month percentage change: GMF and MTR.  2: Baseline T2LV, and BMF, 12 month percentage change average lesion MTR.  3: Baseline age, GMF. 12 month percentage change T2LV. | Outcomes: imputation | Multivariate logistic regression | Discrimination: 1: AUC 0.82. 2: AUC 0.84. 3: AUC: 0.78.  Calibration: absent. Validation: absent. |  |
| Gauthier *et al.* 2007 | Cohort (P)  Single centre  (n=205) | Sustained disability progression | 6 |  | Age, disease duration, EDSS at second study visit, BPF, T2LV | Complete case analysis. | Markov model | Discrimination: absent  Calibration: absent  Validation: absent | Probability curves |
| Held *et al.* 2005 | Placebo arms of clinical trials  Multicentre  (n=821) | Relapse rate | 10 | Stepwise multivariate linear regression selection P-value<0.05. | Relapse number in prior 24 months, disease duration. | Complete case analysis. | Multivariate linear regression. | Discrimination: C statistic NA for continuous outcome  Calibration: absent. Internal validation: split-sample  External validation: absent | Table |
| Liguori *et al.* 2011 | Cohort (R)  Single centre  (n=127) | 1: Change in T2LV  2: Relapse rate  3. Change in BPF | 12 | Stepwise multivariate regression. | 1: First year T2LV change, new/enlarged lesions on first year sMRI, T2LV and BPF at year one.  2: No. CEL at year 0, 1^st^ year T2LV change, resolved / enlarged sMRI lesions.  3: No. of CEL at y1, no. of regressed lesions on y0-y1 sMRI, 1^st^ year change in T2LV. | Complete case analysis | 1: Multivariate linear regression  2: Multivariate negative binomial regression  3. Multivariate linear regression | Overall performance reported R^2^  Calibration: absent.  Validation: absent. |  |
| Mandrioli *et al*. 2008 | Cohort (R)  Single centre  (n=64) | Disability | 13 | Pre-selection by Univariate analysis P-value<0.2 then Cox multivariate analysis (P-value not specified). | CSF IgMOB, sensory onset, pyramidal symptoms, first inter-attack interval. | Complete case analysis. | Multivariate logistic regression | Development: Sensitivity, specificity, PPV and NPV 88.46%, 92.11%, 88.46% and 92.11%, respectively. Discrimination: absent  Calibration: absent  Internal validation: absent  External validation: sensitivity, specificity, PPV and NPV 80.00%, 91.11%, 80.00%, 91.11%, respectively. | Mathematical formula |
| Manouchehrinia *et al.* 2018 | Cohort (P)  Multicentre  (n=8825) | Onset of secondary progression | 15 | Backward selection multivariate regression P-value <0.05. | Age, gender, onset age, first recorded EDSS, age at first recorded EDSS. |  | Multivarite logistic regression | Discrimination: AUC 0.84.  Calibration: calibration plots. Internal validation: bootstrap  External validation: three different cohorts: 1: AUC 0.77. 2: AUC: 0.77. 3: AUC: 0.87 | Nomogram |
| Margaritella *et al.* 2012(A) | Cohort (R)  Single centre  (n=221) | Disability | 8 | Clinical reasoning | Age at onset, gender, course (benign or SP), MEPs, time adjusted mEPs, time adjusted EDSS. | Complete case analysis | Ordinary least squares linear multiple regression | Overall goodness of fit: R^2^: 0.79.  Discrimination: NA continuous outcome.  Calibration: absent.  Validation: absent |  |
| Margaritella *et al.* 2012(B) | Cohort (R)  Single centre (n=143) | Worsening disability | 7 | Bivariate logistic regression P value <0.2. Multivariate regression P-value <0.2. | First EP score and time to EDSS 2 | Complete case analysis. | Multivariate logistic regression | Discrimination: AUC: 0.81. Calibration: not reported.  Internal validation: absent  External: discrimination / calibration not reported. |  |
| Mesaros *et al.* 2008 | Cohort (R)  Multicentre  (n=548) | 1:Disability progression  2: Percentage change cerebral brain volume | 8 | Pre-selection by Univariate regression analysis then multivariate regression. P-value NR. | 1: Baseline EDSS and T2LL.  2: Baseline EDSS, T2LL. | Complete case analysis. | 1: Multivariate logistic regression  2: Multivariate linear regression | Overall performance reported as nagelkerke R^2^. Model 1: 0.03. Model 2: 0.04.  Discrimination: absent.  Calibration: absent.  Internal validation: absent.  External validation: absent. |  |
| Minneboo *et al.* 2008 | Cohort (P)  Single centre  (n=89) | Disability progression | 25 | Forward selection multivariate regression P-value <0.05.  Final model stepwise selection multivariate regression P-value<0.05. | 1: Age, disease type, baseline EDSS.  2 composite of models 1 and PBCV change. | Complete case analysis. | Multivariate logistic regression | Discrimination: 1: AUC: 0.72. 2: AUC: 0.82.  Calibration: not reported.  Internal validation: absent  External validation: absent |  |
| Popescu *et al.* 2013 | Cohort (R)  Multicentre  (n=261) | 1: Disability (EDSS)  2: Disability (MSSS) | 10 | Pre-selection by multivariate analysis P-value<0.05 then backward selection multivariate analysis. | 1: Central atrophy. Lesion volume change.  2: Central atrophy. Lesion volume change. | Complete case analysis. | Multivariate linear regression. | Discrimination: absent  Calibration: absent  Internal validation: absent  External validation: absent |  |
| Ramsaransing *et al*. 2007 | Cohort (P)  Single centre  (n=496) | Disability | 15 | Multivariate logistic regression P-value<0.1 then stepwise multivariate logistic regression. | EDSS at 5 years and number of relapses in the first 5 years. | Complete case analaysis. | Multivariate logistic regression. | Discrimination: absent Calibration: absent Internal validation: absent  External calibration: absent |  |
| Runmarker *et al.* 1994 | Cohort (P)  Single centre  (n=308) | 1: Onset of secondary progression  2: Reaching DSS 6  3: Onset of secondary progression  4. DSS 6 at endpoint  5. Onset of secondary progression | 16 | Multivariate regression P-value <0.10 | 1: Age at onset, gender, remission after first bout, type of affected nerve firbes, first bout mono- or polyregional  2: Age at onset, type of affected nerve fibres, first bout mono- or polyregional.  3. Gender, number of affected neurological systems, remission after last bout, type of affected nerve fibres at last bout, type of affected nerve fibres at last bout. (all collected at year 5).  4. Number of affected neurological systems, remission after last bout, type of affected nerve fibres, type of affected nerve fibres, polyregional symptoms at last bout (all collected at year 5).  5. Age at onset, disease duration, remission after first bout | Complete case analysis | Multivariate Cox regression | Discrimination: absent  Calibration: absent Internal validation: absent External validation: absent |  |
| Schlaeger 2012 | Cohort (P)  Single centre  (n=30) | 1/2a: Disability (EDSS)  2b: Worsened disability | 7 | Bivariate analysis P-value <0.2 then backward selection multivariate regression P-Value <0.2. | Model 1: P100 latency, central motor conduction time, T2 lesions at baseline.  Model 2a: P100 latency and central motor conduction time.  Model 2b: non-linear version of Model 2a. | Imputation | Multivariate linear regression | Model 1: R^2^: 0.40. Model 2a: R^2^: 0.45.  Discrimination: Model 2a AUC 0.76. Model 2b: AUC 0.81.  Calibration: plot presented for Model 2b only. Internal validation: cross-validation External validation: absent |  |
| Schlaeger 2014 | Cohort (P)  Single centre  (n=28) | 1. Disability (EDSS) | 13 | Bivariate analysis P-value <0.2 then backward selection multivariate regression P-Value <0.15. | 1: s-EP (a compound EP measure incorporating P100 latency and central motor conduction time), therapy Y/N. | Imputation. | Multivariate linear regression | R^2^: 0.54.  Discrimination: Model 1: 0.89 for clinical worsening.  Model  Calibration: absent Internal validation: cross-validation  External validation: absent |  |
| Skoog *et al.* 2014 | Cohort (P)  Single centre  (n=157) | Onset of secondary progression (calculates current yearly probability) | 12 | Poisson regression analysis | Current age, afferent symptoms (y/n), complete remission of most recent relapse (y/n), time since last relapse. | Complete case analysis | Poisson regression analysis | Discrimination: absent  Calibration: absent Internal validation: absent External validation: absent | Web based application |
| Sormani et al 2007 | Placebo arm of RCT  Multicentre  (n=539) | Number of relapses at 9 months | 9 | Pre-selection by univariate Cox regression P-value <0.2 then multivariate Cox regression P-value ≤0.01. | Number of relapses in previous two years and number of enhancing lesions retained in final model. | Complete case analysis | Multivariate Cox regression | Discrimination: absent  Calibration: absent  Internal validation: absent  External validation: discrimination / calibration absent | Mathematical formula |
| Uher *et al.* 2017 | Cohort (P) extension of clinical trial  Single centre  (n=181) | Sustained disability progression (2 outcome defintions) | 21 | Pre-selected by univariable regression P-value <0.01 then multivariate Cox regression | T2 lesion volume and CC fraction at baseline, CC % volume change, number of new or enlarging T2 lesions and EDSS absolute change over 12 months. | Complete case analysis | Multivariate Cox regression | Discrimination: absent  Calibration: absent Internal validation: absent  External validation: absent | Risk score |
| Von Gumberz *et al.* 2016 | Cohort (P)  Single cente  (n=109) | 1: Disability change  2: Disability progression dichotomized cutoff | 18 | Linear and logit models then forward stepwise multivariate regression | 1. Baseline EDSS, grey matter volume change and treatment status. | Complete case analysis | 1: Multivariate linear regression  2. Multivariate logistic regression | Overall performance: Model 1 R^2^: 0.29  Discrimination: Model 2: AUC: 0.81.  Calibration: Model 1 reasonable fit. Model 2: absent.  Internal validation: Absent  External validation: Absent |  |
| Weideman *et al.* 2017 | Cohort (P)  Single centre  (n=133) | Disability progression | 12 | Machine learning: gradient boosting machine | Therapy-adjusted CombiWISE/age, measured CombiWISE, COMRIS-CTD, time from disease onset to first therapy, difference between adjusted and unadjusted CombiWISE, age and family history of MS. |  | Machine learning: gradient boosting machine | Overall performance R^2^: 0.65  Discrimination: NA continuous outcome  Calibration: absent  Internal validation: random splitting, R^2^: 0.54.  External validation: absent | Web application |
| Weinshenker *et al.* 1991 | Cohort (P)  Single centre  (n=1099) | Disability | 24 | Multivariate regression accelerated time to failure P-value <0.05 | Gender, age at onset, seen at onset, insidious motor onset, presence of limb ataxia and balance symptoms | Complete case analysis | Multivariate regression accelerated time to failure model | Overall performance presented as log-likelihood.  Discrimination: absent  Calibration: absent  Internal validation: absent  External validation: absent |  |

Cohort (P) = cohort study with prospectively maintained database.

Cohort (R) = cohort study from retrospective data.
