# Supplementary material for: Systematic review of prediction models in relapsing remitting multiple sclerosis
Source: PLoS One. 2020 May 26;15(5):e0233575. doi: 10.1371/journal.pone.0233575 (PMC7250448; doi:10.1371/journal.pone.0233575)
Supplement: S5 File — (DOCX) [file pone.0233575.s006.docx]

**S5 File. Percentage of study participants on DMTs**

| Study | DMTs (%) |
| --- | --- |
| Agosta 2006 | ~60 |
| Bakshi 2008 | 88.34 |
| Barkhof 2005 | 0 |
| Bejarno 2011 | 54.9 |
| Bergamaschi 2001 | 19 glatiramer acetate, <10% on beta-interferon so ~20% |
| Bergamaschi 2007 | 57 |
| Bergamaschi 2015 | 0 |
| De Groot 2009 | 30 |
| Dekker 2019 | 55.7-63.3 |
| Filippi 2012 | 100 |
| Gauthier 2007 | 85.4 |
| Held 2005 | 0 |
| Liguori 2011 | 91.6 |
| Mandrioli 2008 | 65.6 |
| Manouchehrinia 2019 | 100 |
| Margaritella 2012 (A) | NR |
| Margaritella 2012 (B) | 7 |
| Mesaros 2008 | NR |
| Minneboo 2008 | 23.59 |
| Popescu 2013 | 47.5 |
| Ramsaransing 2007 | 14.5-21.5 |
| Runmarker 1994 | 0 |
| Schlaeger 2012 | 72 |
| Schlaeger 2014 | 75 |
| Skoog 2014 | 0 |
| Sormani 2007 | 0 |
| Uher 2017 | 100 |
| Von Gumberz 2016 | 36 |
| Weideman 2017 | NR |
| Weinshenker 1991 | 0 |

NR = not recorded
