# Supplementary material for: Systematic review of prediction models in relapsing remitting multiple sclerosis
Source: PLoS One. 2020 May 26;15(5):e0233575. doi: 10.1371/journal.pone.0233575 (PMC7250448; doi:10.1371/journal.pone.0233575)
Supplement: S6 File — (DOCX) [file pone.0233575.s007.docx]

**S6 File. Events per variable per study and calculation method.**

Events / variables

Events = as determined by the smaller of the two groups: with or without outcome.

Variables = the sum of number of regression coefficients of *candidate* predictors. As per PROBAST guidance.

***Events per variable per study (development only)***

| Study | EPV |
| --- | --- |
| Agosta 2006 | 1.16 |
| Bakshi 2008 | 6 |
| Barkhof 2005 | 62.4 |
| Bejarno 2011 | 0.78, 1.105 |
| Bergamaschi 2001 | 1.88 |
| De Groot 2009 | 1.6 |
| Dekker 2019 | NA |
| Filippi 2012 | 1.65, 0.52, 0.52, 1.13 |
| Gauthier 2007 | NR |
| Held 2005 | NA |
| Liguori 2011 | NA |
| Mandrioli 2008 | 2.0 |
| Manouchehrinia 2019 | 78.31 |
| Margaritella 2012 (A) | NA |
| Margaritella 2012 (B) | NR |
| Mesaros 2008 | 10 |
| Minneboo 2008 | 1.44 |
| Popescu 2013 | NA |
| Ramsaransing 2007 | 9.43 |
| Runmarker 1994 | NR |
| Schlaeger 2012 | NA |
| Schlaeger 2014 | NA |
| Skoog 2014 | 9.83 |
| Sormani 2007 | NR |
| Uher 2017 | 4.90, 3.04 |
| Von Gumberz 2016 | 1.11 |
| Weideman 2017 | NA |
| Weinshenker 1991 | 20.75 |

NA = not applicable in model with continuous outcome

NR = insufficient data reported to calculate
